# Cardiovascular Physiotherapy on Respiratory Sinus Arrhythmia of Patients Undergoing Coronary Artery Bypass Grafting

**DOI:** 10.21470/1678-9741-2020-0276

**Published:** 2021

**Authors:** Bianca Lopes Silva, Roberto Ribeiro da Silva, Hugo Valverde Reis, Ana Carolina Accosio Rodriguez, Priscila Souza e Souza, Isabela de Andrade, Leonardo Fonseca, Solange Guizillini, Michel Silva Reis

**Affiliations:** 1 Faculty of Physiotherapy, Research Group in Cardiorespiratory Evaluation and Rehabilitation (GECARE), Federal University of Rio de Janeiro (UFRJ), Rio de Janeiro, RJ, Brazil.; 2 Graduate Program in Physical Education, Federal University of Rio de Janeiro (UFRJ), Rio de Janeiro, RJ, Brazil.; 3 Graduate Program in Cardiology, Federal University of Rio de Janeiro (UFRJ), Rio de Janeiro, RJ, Brazil.; 4 Federal Hospital of State Public Servants, Rio de Janeiro, RJ, Brazil.; 5 Federal University of São Paulo (UNIFESP), São Paulo, SP, Brazil.

**Keywords:** Heart Rate Variability, Respiratory Sinus Arrhythmia Maneuver, Cardiovascular Physiotherapy, Coronary Artery Bypass Grafting

## Abstract

Introduction: Patients in the postoperative period of coronary artery bypass grafting (CABG) present respiratory and autonomic dysfunctions. In this sense, cardiovascular physiotherapy has been offered as an indispensable differential for the improvement of the prognosis of this population. Heart rate variability is a simple, noninvasive method to analyze autonomic modulation, as well as the accentuation maneuver of respiratory sinus arrhythmia, which demonstrates the parasympathetic autonomic control over the heart. Five patients undergoing cardiac surgery performed a protocol of cardiovascular physiotherapy in the postoperative period and had their data referring to the preoperative period, the 1st and 4th postoperative days analyzed.

**Table:** 

Abbreviations, acronyms & symbols	
**ANS**	**= Autonomic nervous system**
**CABG**	**= Coronary artery bypass grafting**
**CP**	**= Cardiovascular physiotherapy**
**ECG**	**= Electrocardiogram**
**HR**	**= Heart rate**
**HRV**	**= Heart rate variability**
**M-RSA**	**= Maneuver of accentuation of respiratory sinus arrhythmia**
**RMSSD**	**= Root mean square of the successive differences**
**RRi**	**= R-R interval**
**RSA**	**= Respiratory sinus arrhythmia**
**SDNN**	**= Standard deviation of NN intervals**

## INTRODUCTION

Cardiovascular physiotherapy (CP) is an integral part of the care of patients in the postoperative period of coronary artery bypass grafting (CABG), since it contributes to a better prognosis regarding the recovery of autonomic and respiratory disorders present in the period. In this context, the autonomic heart rate (HR) modulation can be evaluated in a non-invasive way, based on the analysis of heart rate variability (HRV) and HR response^[1,2]^ in autonomic function tests, as in the maneuver of accentuation of respiratory sinus arrhythmia (M-RSA)^[3,4]^.

Respiratory sinus arrhythmia (RSA) is a physiological event characterized by HR oscillations associated with the respiratory cycle^[5]^. Such phenomenon seems to occur by an inhibition of parasympathetic activity during inspiration, with M-RSA being a tool to evaluate this autonomic control. Reduction in HRV and RSA may be associated with abnormal and insufficient autonomic nervous system (ANS) adaptability over cardiac autonomic control of patients after CABG and have an influence on their prognosis^[1,6]^. Pulmonary dysfunction, associated with respiratory complications, such as the need to remain with invasive or non-invasive ventilatory support for longer than scheduled, atelectasis and hypoxemia, increase the rate of morbidity and mortality in this period^[7,8]^.

Currently, few studies contemplate the HRV analysis in the postoperative period of CABG with the application of an exercise-based CP protocol, and none of them provides complementary data on the behavior of RSA in this period. However, the analysis of this variable brings information about the integrity of parasympathetic modulation and how the ventilatory mechanics can alter this modulation^[9,10]^. In view of this, our objective was to evaluate the effect of CP on cardiac autonomic modulation during the postoperative period of cardiac surgery.

## COMMENTS

Five patients undergoing CABG were admitted to the Coronary Care Unit of the Federal Hospital of State Public Servants of Rio de Janeiro. They performed a CP protocol in the postoperative period and had their data referring to the preoperative period, the 1^st^ and 4^th^ postoperative days analyzed. This study was approved by the Ethics Committee of Federal Hospital of State Public Servants of Rio de Janeiro (CAEE: 39101114.2.0000.5257). Initially, the patients underwent two types of daily evaluations: (i) Clinical cardiological evaluation performed daily by the unit's physician. This assessment consisted of routine tests to characterize the clinical evolution, arterial blood gas analysis, laboratory tests (blood count, biochemistry, electrolytes) and electrocardiogram; (ii) Physiotherapy evaluation: consisting of anamnesis and physical examination, to investigate the history and current illness, as well as life and eating habits. The evaluations were performed daily, always at the same time of day, considering circadian variations, to define their progression or not in the experimental protocol.

CP consisted of: (i) 1^st^ postoperative day - Head of bed elevated 45º: breathing exercises (4 min) and active-assisted exercises of upper and lower limbs - 3 sets of 10 repetitions; (ii) 2^nd^ postoperative day: Patient seated at 90º: breathing exercises (4 min) and active-free exercises of upper and lower limbs (2 sets of 15 repetitions). Orthostatic posture: stationary walking (5 min or limited by symptoms); 3^rd^ postoperative day: Seated patient: breathing exercises and repetition of stage 2 with 3 sets of 15 repetitions. Ambulation in the corridor (5 min); 4^th^ postoperative day: Seated patient: repetition of stage 3. Ambulation in the corridor (10 min); 5^th^ postoperative day: Patient in orthostatic position: repeat stage 3. Ambulation in the corridor (10 min) and up and down stairs (4 steps).

HR and R-R intervals (RRi) were recorded beat by beat using a heart rate monitor (Polar^®^RS800). While using this heart rate monitor, data was transmitted simultaneously to the watch where it was stored. The collection was performed at rest in the supine position for 10 minutes. The same heart rate monitor was used during the performance of the M-RSA, when the patient was instructed, by verbal and tactile command (abdominal stimulation), to perform deep and slow nasal inspirations and oral expirations, varying the pulmonary volume from total lung capacity to residual volume, according to the existing protocol^[5]^. HRV data were analized using Kubius HRV 2.0 application for Windows. Initially, from the visual inspection, the 5-minute stretches with the highest stability of the resting ECG RRi tracing were selected. Time domain analysis was performed by means of HR, mean RRi, standard deviation of NN intervals (SDNN) and root mean square of the successive differences (RMSSD) indexes^[1]^. Then, through a specific routine developed in the MATLAB application, the following HR and RRi indexes obtained from M-RSA were calculated: expiration/inspiration ratio (E/I) and inspiration-expiration difference (ΔIE)^[3]^. All patients who participated in the study started the protocol from the 1^st^ postoperative day after orotracheal extubation and with medical consent and performed the activities once a day for at least five days of hospitalization according to Table 1^[10]^.

**Table 1:** Anthropometric data and clinical characteristics of the patients.

Variables	Patients (n=5)
Age (years)	66.4±10
Gender (M/F)	03/02
Height (m)	1.6±1
Body mass (kg)	76.1±10
BMI (kg/m^2^)	29.5±6
**Clinical features**	
Resting HR (bpm)	63.6±10
Resting SBP (mmHg)	110±12
Resting DBP (mmHg)	66.8±6
SpO_2_	96.8±1
**Lesion (n/%)**	
Bivascular	2/40
Multivascular	3/60
**Risk factors (n/%)**	
SAH	5/100
DM	1/20
Dyslipidemia	3/60
Positive FH	4/80
**Surgical data**	
CPB (n/%)	2/40
CPB (min)	35.8±50
MV (min)	178±273
**Drug treatment (n/%)**	
Beta blocker	4/80
ACEi	3/60
Diuretics	4/80

Values expressed as mean ± standard deviation. ACEi=angiotensin-converting-enzyme inhibitors; BMI=body mass index; CPB=cardiopulmonary bypass; DBP=diastolic blood pressure; DM=diabetes mellitus; FH=family history; HR=heart rate; HT: hypertension; MV=mechanical ventilation; SBP=systolic blood pressure; SpO_2_=peripheral oxygen saturation

**Fig. 1:**
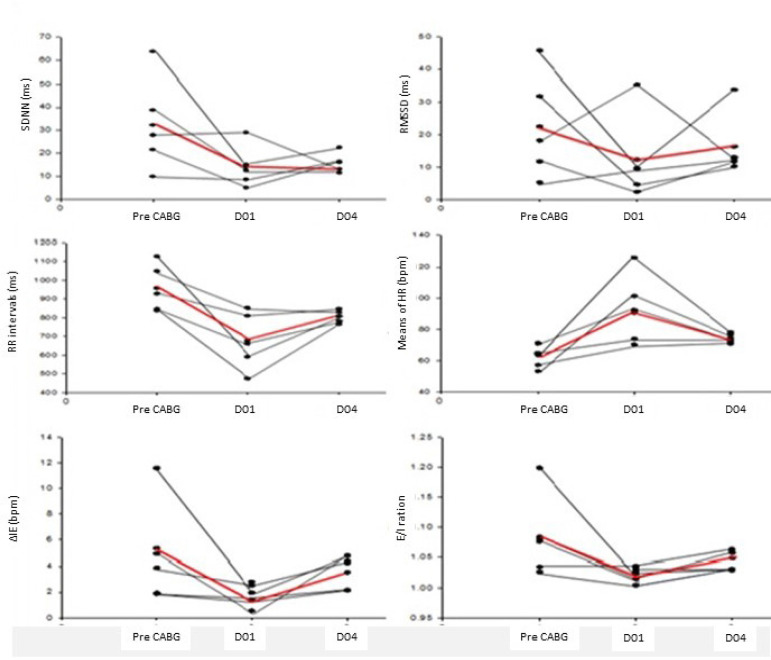
Heart rate variability and maneuver of respiratory sinus arrhythmia pre- and post-CABG. HRV and amplitude of RSA values during M-RSA of the five volunteers. ▬ Mean; ▬ Patient. ΔIE=difference between the mean of the highest HR values obtained during the inspiratory phase and the mean of the lowest HR values during the expiratory phase of M-RSA; D01=1st postoperative day; D04=4th postoperative day; E/I ratio=mean of the highest RRi values obtained during the expiratory phase divided by the means of the lowest RRi values of the inspiratory phase of the M-RSA; HR=heart rate; HRV=heart rate variability; M-RSA=maneuver of accentuation of respiratory sinus arrhythmia; RMSSD=square root of the mean square of successive differences between adjacent RRi divided by the number of RRi minus one in ms; RRi=R-R intervals; SDNN=standard deviation of all RRi in ms

The data were analyzed by descriptive statistics organized in Sigmaplot (version 11 for Windows). The results were presented as mean and standard deviation. The anthropometric data and general characteristics of the individuals were shown in Table 1. HRV and M-RSA values were shown in Figure 1.

The main findings of this study are that HRV indexes of post-CABG patients undergoing CP showed improvement in the index related to parasympathetic modulation (RMSSD). Additionally, on the 4^th^ postoperative day, most patients had values like those in the preoperative period. Interestingly, during the M-RSA, the values that represent the amplitude of the RSA showed a reduction, improving in the 4^th^ postoperative day. It is already consolidated in the literature that, immediately after CABG, the autonomic dysfunction is present and this dysfunction is related to a greater number of cardiovascular complications, worse prognosis and higher mortality^[6,7,9,10]^.

Soares et al.^[11]^ (2005) showed that cardiac autonomic modulation worsens substantially in the first six postoperative days and the reversibility of this worsening occurs only 60 days after CABG. However, in our study, for four days a behavior of return to the basal values could already be observed in some important variables such as HR and SDNN index. Some studies^[(11,12]^ have already demonstrated that physical exercise during the postoperative period of CABG has several benefits in autonomic modulation.

Takeyama et al. (2000)^[13]^ showed that physical exercise during the postoperative period of CABG improves functional capacity and parasympathetic modulation. Therefore, analyzing the autonomic modulation in the postoperative period of CABG would give us the possibility to evaluate the improvement of this system during exercise application, contributing to the standardization of CP prescription, in addition to improved safety and efficiency^[8]^.

Pulmonary function impairment after CABG is expected and has several causes, such as: sternotomy, pain, pleurotomy by graft incision^[14]^, pleural drainage insertion site^[7,14,15]^, diaphragmatic dysfunction, general anesthesia and reflex dysfunction of the phrenic nerve^[8,16,17]^, in addition to the use of cardiopulmonary bypass^[16,17]^. Changes in pulmonary mechanics, restrictive respiratory pattern, and shallow breathing are commonly observed in these patients. In this context, a variety of respiratory maneuvers were the basis of CP care in post-CABG patients. However, early mobilization has proved to be a central strategy in the management of patients undergoing this procedure^[18,19]^.

Data analyzed during M-RSA are unpublished so far in this population. We believe that the improvement in the amplitude of M-RSA is probably related to the fact that these patients, in addition to improving autonomic modulation, also improved pulmonary function with inclusion in CP care. Respiratory exercises and mobilization are able to increase tidal volume, which in turn directly alter the M-RSA amplitude data. The improvement of pulmonary dysfunction provides these patients early cardiopulmonary reconditioning to perform their daily life activities, to generate better prognoses and less possibility of new cardiovascular events^[7,10,16-19]^.

The limiting conditions of the present study are the sample size, the difficulty in keeping the protocol days unchanged and the lack of daily data, allowing a more precise follow-up of what happened during the entire hospitalization.

## CONCLUSION

In conclusion, it was possible to observe better values of autonomic modulation close to the hospital discharge period, suggesting that the use of an exercise-based cardiovascular physiotherapy protocol may have favored this finding. The indexes that correspond to vagal modulation were the most expressive. The amplitude of M-RSA, described for the first time in this population and during the intervention, also showed values suggestive of improvement close to the 4^th^ postoperative day.

**Table:** 

Authors' roles & responsibilities	
BLS	Substantial contributions to the conception or design of the work; or the acquisition, analysis, or interpretation of data for the work; final approval of the version to be published
RRS	Acquisition, analysis, or interpretation of data for the work; final approval of the version to be published
HVR	Substantial contributions to the conception or design of the work; or the acquisition, analysis, or interpretation of data for the work; final approval of the version to be published
ACAR	Acquisition, analysis, or interpretation of data for the work; final approval of the version to be published
PSS	Acquisition, analysis, or interpretation of data for the work; final approval of the version to be published
IA	Final approval of the version to be published
LF	Drafting the work or revising it critically for important intellectual content; agreement to be accountable for all aspects of the work in ensuring that questions related to the accuracy or integrity of any part of the work are appropriately investigated and resolved; final approval of the version to be published
SG	Drafting the work or revising it critically for important intellectual content; agreement to be accountable for all aspects of the work in ensuring that questions related to the accuracy or integrity of any part of the work are appropriately investigated and resolved; final approval of the version to be published
MSR	Drafting the work or revising it critically for important intellectual content; agreement to be accountable for all aspects of the work in ensuring that questions related to the accuracy or integrity of any part of the work are appropriately investigated and resolved; final approval of the version to be published
